# Investigating the Effects of Language-Switching Frequency on Attentional and Executive Functioning in Proficient Bilinguals

**DOI:** 10.3389/fpsyg.2020.01078

**Published:** 2020-07-08

**Authors:** Cristina-Anca Barbu, Sophie Gillet, Martine Poncelet

**Affiliations:** Psychology and Neuroscience of Cognition Research Unit, University of Liège, Liège, Belgium

**Keywords:** language-switching frequency, bilingualism, attentional and executive functioning, alerting, response inhibition, cognitive flexibility

## Abstract

Recent studies have proposed that the executive advantages associated with bilingualism may stem from language-switching frequency rather than from bilingualism *per se* (see, for example, Prior and Gollan, 2011). Barbu et al. (2018) showed that high-frequency switchers (HFLSs) outperformed low-frequency switchers (LFLSs) on a mental flexibility task but not on alertness or response inhibition tasks. The aim of the present study was to replicate these results as well as to compare proficient (HFLSs and LFLSs) to a control group of monolingual participants. Two groups of proficient bilingual adults (30 HFLSs and 21 LFLSs) and a group of 28 monolinguals participated in the study. The results showed superior mental flexibility skills in HFLSs compared to (LFLSs) and monolinguals; furthermore, the two latter groups showed no difference in mental flexibility skills. These results provide novel support for the hypothesis that the so-called bilingual advantage is, in fact, a result of language-switching habits.

## Introduction

Assessing the cognitive effects of bilingualism has been an important scientific issue since the early 1920s. At this time, the general consensus in the psycholinguistics field was that learning a second language (L2) had a negative effect on cognitive development, affecting skills such as verbal and non-verbal intelligence, arithmetic, and reading (Graham, 1925; Wang, 1926; Darcy, 1963). This vision started to change in the 60s when Peal and Lambert (1962) reported data for the first time showing that bilingualism does not engage negative effects on non-verbal or verbal intelligence; rather, it improves these skills. Negative results observed before 1962 have been attributed to a series of methodological flaws, as these studies did not control for different factors including L2 type, level of bilingualism, and socio-cultural status. These factors have been shown to influence results and are likely to represent underlying factors for the observed effects. For instance, when bilinguals’ language knowledge is assessed and participants’ intelligence skills are tested in the stronger language and not in the weaker L2, no significant differences between bilinguals and monolinguals are observed, and advantages in favor of bilinguals are even detected (for a review, see Darcy, 1963; Hakuta, 1986).

Starting with Peal and Lambert’s (1962) study, several authors began to report that bilingualism has a positive effect on cognition, affecting in particular attentional and executive functioning (for a review, see Bialystok, 2011; Dong and Li, 2015). These advantages have been observed on different attentional and executive skills including alertness (e.g., Costa et al., 2008), interference and response inhibition (e.g., Costa et al., 2008, 2009; Fernandez et al., 2014), and cognitive flexibility (e.g., Prior and Gollan, 2011; Ibrahim et al., 2013; Liu et al., 2015). Further advantages have been shown among different bilingual populations, including children (e.g., Bialystok and Barac, 2012; Nicolay and Poncelet, 2013, 2015; Kalashnikova and Mattock, 2014), young adults, middle-aged adults, and even older-age adults (e.g., Bialystok et al., 2014). These benefits have been generally attributed to the continual transferring of different linguistic structures from one language to another during language learning (e.g., Costa et al., 2016) and to the ongoing need of bilinguals to inhibit one of their two activated languages (e.g., Green, 1998).

Recent research in this respect has, however, revealed that the finding of cognitive benefits may not be replicated in a consistent manner (e.g., Paap and Greenberg, 2013). This lack of consistency has been ascribed to different non-controlled factors, including L2 proficiency, L2 onset age, and language-switching frequency. Bilinguals can effectively differ on these different linguistic aspects, which can influence subjects’ performance of tasks assessing attentional and executive functioning (e.g., Lehtonen et al., 2018). Other non-controlled factors, including socio-cultural status, video game practice, and music practice have also been shown to influence attentional and executive functioning (Boot et al., 2008; Brito and Noble, 2014; Hackman et al., 2015).

Language-switching frequency (e.g., Dong and Li, 2015) has been proposed as being a responsible factor for bilingual advantages in tasks assessing executive functioning. Switching between languages occurs in two types of situations: when a bilingual switches from one language to the other one with another bilingual or when the person switches from first language (L1) to L2 (or vice versa) to adapt to the language of the monolingual interlocutor.

Despite the expansive interest in the cognitive effects of this linguistic behavior, relatively few studies have investigated the effect of language-switching frequency on attentional and executive functioning in bilinguals (Prior and Gollan, 2011; Hartanto and Yang, 2016; Verreyt et al., 2016; Barbu et al., 2018). These studies have revealed globally that language-switching frequency has a positive effect on cognitive flexibility and interference inhibition (Prior and Gollan, 2011; Hartanto and Yang, 2016; Verreyt et al., 2016; Barbu et al., 2018) but not on alertness or response inhibition skills (Barbu et al., 2018).

For instance, Verreyt et al. (2016) showed that language switching has a positive impact on interference inhibition skills in proficient bilinguals. In this study, performances of unbalanced and balanced Dutch/French-speaking bilingual adults were compared on the Attention Network Test (ANT) (Fan et al., 2002), a measure of interference inhibition. Balanced bilinguals included high- and low-frequency language switchers (HFLSs and LFLSs). During this task, participants are presented with five arrows appearing in the middle of the computer screen. The central arrow (the target) pointing left or right is surrounded by arrows (flankers) pointing either in the same direction (congruent condition) or in the opposite direction (incongruent condition) as the target. A control condition is also available in which the target arrow is surrounded by bars. Participants are instructed to press a response key (e.g., right or left) as fast as possible depending on the direction of the target. Differences in response speed between congruent and incongruent conditions (conflict effect) are recorded. Results revealed that (HFLSs) bilinguals exhibited a more reduced conflict effect as compared to (LFLSs) bilinguals and (LFLSs). No group difference was observed between the low-frequency switchers and low-proficient bilinguals. This advantage was attributed to the bilingual ability to switch actively between languages. The authors argued that language-switching frequency enhances resistance to distractor interference in bilingual adults, as these skills are required in order to prevent intrusions from the non-intended language.

Prior and Gollan (2011) have also revealed a positive effect of language-switching frequency on executive functioning but this time on general shifting (cognitive flexibility skills). In this study, proficient Spanish–English bilinguals switching frequently between languages were compared to proficient Mandarin–English bilinguals switching rarely between the two and English monolinguals on a measure assessing switching skills. This measure consisted of a non-linguistic as well as a linguistic switching task, both based on the same experimental design. In the non-linguistic task, participants were asked to perform color and shape judgments on visual stimuli (red or green circles and triangles) presented on a computer screen. In the linguistic version, participants were asked to name digits (from 1 to 9) as fast as possible in their L1 and L2. For both tasks, two measures were recorded: switch costs (the mean response speed difference between task-switch and task-repeat trials in mixed-task blocks) and mixing costs (the mean response speed difference between mixed-task trials and task-repeat trials within single-task blocks). Results revealed no group difference on mixing costs in either task. However, a significant group difference was observed in terms of switch costs on both the linguistic and non-linguistic tasks, with high-switching Spanish–English bilinguals outperforming low-switching Mandarin–English bilinguals and English monolinguals. No significant group difference was observed in this respect between the later two groups. This advantage was again attributed to language-switching frequency. According to the authors, language-switching frequency improves task-switching skills (involved in the switching task applied) given that both language switching and task switching rely on similar requirements (switching between mental sets) and both are based on a common process of general switching skills.

Hartanto and Yang (2016) also observed similar findings (no group differences on correct responses or mixing costs but a significant group difference on switch costs) by comparing two groups of proficient bilinguals (i.e., HFLSs and LFLSs). They used a similar switching task (requiring subjects to switch between color and shape trials) to assess switching skills. Rather than linking this advantage to the bilingual ability to switch actively between mental sets, the authors stated that this benefit should rather be attributed to the improved bilingual ability to inhibit intrusions from the non-target language when switching between languages.

In line with these findings, Barbu et al. (2018) also revealed that language-switching frequency enhances cognitive flexibility skills in bilingual adults. The authors compared two groups of proficient bilingual adults with different language-switching patterns, i.e., HFLSs and LFLSs, on a series of attentional and executive tasks assessing alertness, response inhibition, and cognitive flexibility. The results revealed a small group difference (*p* = 0.03), with high-frequency switchers outperforming low-frequency switchers in terms of response speed on the cognitive flexibility task. No significant group differences were, however, observed on the alertness and response inhibition measures. The authors determined that language-switching frequency enhances cognitive flexibility, given that they both require mental shifting, behavior which would indirectly improve non-verbal general switching skills. Concerning the lack of between-group differences observed on the alertness and response inhibition tasks applied, the authors suggested that that these advantages were not observed given that the tasks used to assess these skills did not require a behavior similar to language switching, i.e., switching between mental sets.

Alertness and response inhibition skills may be enhanced not by language-switching frequency but by bilingualism itself. Considering that the two bilingual groups (HFLSs and LFLSs) tested by Barbu et al. (2018) had the same L2 proficiency levels, no significant group differences were observed in this respect given that these skills were probably used to the same extent as subjects became bilinguals.

The aim of the present study was to replicate Barbu et al.’s (2018) data by testing HFLSs and LFLSs with homogenous language backgrounds (only speakers of German and French). This research also compares a performance of these two groups to a monolingual control group on tasks assessing alertness, response inhibition, and cognitive flexibility. If language-switching frequency is a specific factor that enhances mental flexibility skills, high language switchers should outperform low language switchers and monolinguals. However, the later two groups should not differ in this respect. If bilingualism is also a contributing factor to this advantage, low language switchers should exhibit a better performance than monolinguals. Concerning the alertness and response inhibition tasks, given the results of Barbu et al. (2018), we expect to find no significant group difference between high and low language switchers. If bilingualism in itself produces a cognitive benefit, high and low language switchers should outperform monolinguals.

## Materials and Methods

### Participants

A total of 79 participants were recruited for this study. This included two groups of bilinguals composed of 30 HFLS and 21 LFLS speakers of German and French. In addition, 28 French-speaking monolinguals were recruited for this study. HFLSs, LFLSs, and monolinguals had no psychological, auditory, or language deficits at the time of testing. None of the participants were involved in professional activities including intensive sports or music training.

Part of the assessed bilingual population (13 HFLSs and 6 LFLSs) were recruited from a cohort tested by Barbu et al. (2018). HFLSs and LFLSs were assigned to their corresponding groups according to their language-switching frequency rates provided by means of a language questionnaire. In order to assess language-switching frequencies, HFLSs and LFLSs were asked to rate and total the times they orally switched between languages on a weekly basis. This total number was divided by seven in order to establish participants’ daily language-switching rates. In order to determine the effects of language-switching frequency on attentional and executive functioning, we selected only bilinguals with contrasting language-switching frequency rates: 30 HFLSs switching orally between languages from 20 to 120 times on a regular daily basis (i.e., mean language-switching frequency: 43.29 ± 22.34) and 21 LFLSs switching orally between languages from 0 to 6 times per day (i.e., mean language-switching frequency: 3.65 ± 2.17). HFLSs and LFLSs were selected from a large pool of 68 French–German and German–French bilingual speakers who switched from 0 to 120 (mean frequency rate: 22 switches per day). These participants had a high level of proficiency in L2, as estimated by self-rated L2 skills in speaking and speech comprehension, and all had French and German as either their L1–L2 or L2–L1 languages. The 17 remaining bilinguals who switched between 7 and 18 times per day were excluded from the analysis. HFLSs and LFLSs had a similar level of L2 proficiency, as reported by self-rated L2 skills in speaking, reading, writing, and speech comprehension and by an assessment of receptive L2 vocabulary skills using an adaptation of the British Picture Vocabulary Test (BPVT) (Dunn et al., 1982), a productive vocabulary measure (Cardebat et al., 1990), and a general vocabulary knowledge measure, Lexical Test for Advanced Learners of English (LexTALE; Lemhöfer and Broersma, 2012; Brysbaert, 2013). All measures were adapted in French or German according to participants’ L2. HFLSs and LFLSs used their L2 to a similar extent as shown by self-estimated weekly L2 frequency of use and were matched in terms of L1–L2 language membership.

HFLSs and LFLSs were also matched in terms of third language (L3) proficiency skills as shown by self-reported L3 proficiency skills in speaking, reading, writing, and speech comprehension and by a self-reported weekly L3 frequency use. All three language groups (HFLSs, LFLSs, and monolinguals) were matched in terms of L1 language proficiency levels as shown by self-reported L1 proficiency skills in speaking, reading, writing, and speech comprehension. The measures included an L1 receptive vocabulary measure, the Peabody Picture Test (Dunn et al., 1982), an L1 productive vocabulary measure (Cardebat et al., 1990), and an L1 general vocabulary knowledge measure, LexTALE (Lemhöfer and Broersma, 2012; Brysbaert, 2013). All measures were adapted in French or German according to participants’ L1. These groups used their L1 to a similar extent on a regular daily basis as shown by self-reported weekly L1 frequency of use.

The high-frequency language group (HFLS) was composed of 24 women and 6 men ranging between 18 and 39 years (M = 25.73, SD = 6.08). In this group, 23 participants spoke German as their L1 and French as their L2. Six participants used French as their L1 and German as their L2, and one reported having French and German as L1. HFLSs mastered various L3s including English (25), Dutch (3), and Spanish (1). One participant reported having no L3 language knowledge.

The low-frequency language group (LFLS) was composed of 19 women and 2 men ranging in age between 19 and 44 years (M = 24.90, SD = 6.65). In this group, 15 participants spoke German as their L1 and French as their L2. Six participants used French as their L1 and German as their L2. Participants mastered several L3 including English (20) and Dutch (1).

The monolingual group consisted of 23 women and 5 men ranging in age from 20 to 44 years (M = 27.89, SD = 7.16). Monolinguals listed French as their L1 most mastered and the language used at the time of testing as revealed by self-rated L1 skills in speaking, reading, writing, and speech comprehension. They also self-rated weekly L1 frequency of use. Moreover an assessment of receptive L1 vocabulary skills was conducted via the BPVT test adapted to French (Dunn et al., 1982). Monolinguals’ L1 proficiency skills were also assessed using a productive vocabulary measure (Cardebat et al., 1990) and a general vocabulary measure, LexTALE (Brysbaert, 2013), both adapted to French. These participants also mastered an L2 (English), although to a low level, and they were rarely using this language as indicated by an English receptive vocabulary measure, the BPVT (Dunn et al., 1982), and by a self-reported weekly L2 frequency use. In order to assess subjects with homogenous language pairs, we selected only monolinguals with French as L1 and English as L2. These subjects were considered monolinguals provided that they rated themselves as having a maximum basic English oral productive level on self-reporting oral productive Likert scales and scored at least −2 SD on the BPVT test.

Participants did not receive any course credit or payment for their participation.

### General Control and Language Measures

#### General Control Measures

##### Video game practice, socio-cultural status, and non-verbal intelligence skills

Given that intensive ***video gaming*** and high ***socio-cultural status*** have also been shown to enhance attentional and executive functioning (e.g., Castel et al., 2005; Verburgh et al., 2014; Zuk et al., 2014; da Rosa Piccolo et al., 2016), we controlled for these factors. Video gaming was assessed by asking participants to estimate their weekly practice time. In order to determine participants’ SES levels, they were asked to rate the total number of years of study they completed since first grade.

***Non-verbal intelligence skills*** were assessed by using Ravens’ Progressive Matrices (Raven et al., 1982). In this task, participants were required to identify which one of a series of proposed segments best completed a large visual–spatial pattern. Participants were given a maximum of 20 min’ time to perform the task. The total correct responses were recorded and used in the analysis.

#### Language Measures

***Receptive vocabulary skills*** were measured by using different versions of the Peabody test (Dunn et al., 1982) adapted in German (Dunn and Dunn, 1997), French (Dunn et al., 1993), and English (BPVT: Dunn et al., 1982). In all of the versions, participants were shown four images on a computer screen and asked to indicate the image that best corresponded with the word spoken by the administrator. Items were ordered by increased levels of difficulty. Testing procedures were applied according to test instructions. The total correct responses were recorded as an indication of test performance. In order to assure the comparability of the different test versions, raw scores were converted into standard scores (z scores) and used in the analysis.

***Productive vocabulary skills*** were assessed by using different versions of a verbal fluency task (Cardebat et al., 1990), adapted in French and German. A German version of the test adapted according to Tucha et al. (2000) was specifically created for this study. The two French and German test versions were identical in terms of the total number of items proposed and the testing procedures: Participants were given 2 min and required to produce orally as many words as possible starting with a specific phoneme (P, R, V in French and B, M, L in German) or belonging to a specific semantic category (animals, fruits, and furniture for both German and French versions). They were instructed to avoid giving proper nouns or items belonging to the same language family (e.g., grandfather, grandpa, great grandfather). Total correct responses were recorded and introduced in the analysis.

***General vocabulary skills*** were measured by a written lexical decision task adapted in French (LexTALE: Brysbaert, 2013), German, and English (Lemhöfer and Broersma, 2012). Testing procedures were the same for all three test versions. These versions differed in terms of the total number of items proposed. During these tasks, written letter sequences were presented to participants on paper. They were required to identify only sequences corresponding to real words. A global accuracy score was established by calculating the mean percentage of correct responses for words and pseudo-words. This score was used in the analysis.

##### Self-estimated L1, L2, and L3 proficiency skills

Participants’ L1, L2, and L3 proficiency levels were assessed by using a six-point Likert scale in speaking, reading, writing, and speech comprehension (from 1 = very low to 6 = very high).

### Experimental Mesures

Different measures for***alertness, cognitive flexibility*, and *response inhibition*** were assessed from the Test of Attentional Performance battery (TAP) (Zimmermann and Fimm, 2009), a computerized *standardized* battery used to evaluate different attentional aspects. *For each of these tasks, only one condition assessing the target function was available so that we couldn’t compare different conditions in this task.* A detailed description of the tasks employed is presented below:

***Alertness*** was measured by using the alertness subtest of the TAP battery. Participants were required to press a key response as fast as possible when a visual stimulus (an “x” sign) appeared in the center of the computer screen. The task consists of 20 trials of which the first two are dummies. Reaction times and aberrant responses or errors (=RT superior to mean + 2.35 × standard deviation) were recorded.

***Response inhibition*** was measured using the Go/NoGo subtest from the TAP battery. Participants were asked to press a key response as quickly as possible when an “x” sign appeared in the middle of the computer screen and to withhold their answer when a “+” sign was present. The tasks included 40 trials (20 targets «x» and 20 distractors «+»). Each stimulus (distractor or target) was presented for a maximum of 200 ms. Reaction times and errors were recorded.

**FIGURE 1:**
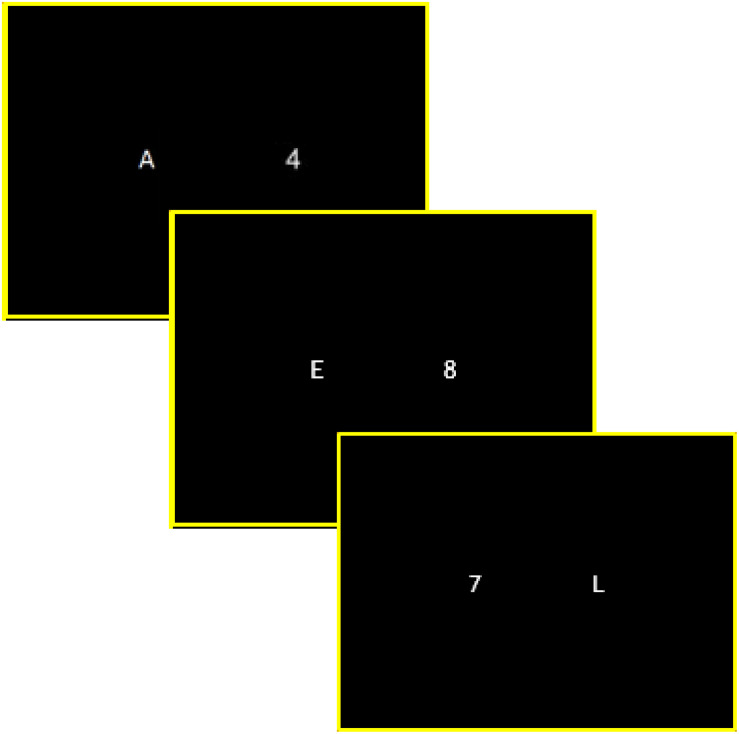
Example of trials proposed during the cognitive flexibility task. For each trial, participants are presented with two stimuli (one on the right side and the other on the left side of the computer screen) and are asked to determine the position of the target item (letter or digit) by pressing the corresponding response key located on the right or left side of the keyboard. First, participants are required to respond depending on the position of the letter and then for the position of the digit and then to alternate between the two as fast and as accurately as possible. For instance, for the first trail presented on-screen (A, left side, and 4, right side) participants are first required to press the left key according to the letter position (A on the left side). For the second trail (E, left side, and 8, right side) participants are first required to press the right key according to the digit position (8 on the right side) and so forth.

***Cognitive flexibility*** was measured using the cognitive flexibility subtest of the TAP battery. In this task, a pair of stimuli (a letter and a number) appears randomly on the right or left side of the computer screen. Participants were required to determine (by pressing a right or left response key) the position (right or left) of a target item (either letter or number) and then to alternate between the two. First, participants were asked to respond according to the position of the letter and then for the position of the number, and so forth. The position of the target stimulus could not be foreseen (see Figure 1 for an exemple). Acoustic feedback was given when errors were made. The task was comprised of a total of 100 items and lasted approximately 3.5 min. Reaction times and errors were recorded.

### General Procedures

All participants were tested in French in an individual session, which lasted from 3 to 4 h (depending on their speed of task resolution). Testing began with the administration of L2 proficiency tasks (receptive, productive, and general knowledge tasks). The testing session continued with the application of the attentional and executive tasks from the TAP battery, followed by the L1 and L3 proficiency tasks (receptive, productive, general knowledge tasks) and a non-verbal intelligence task. Participants were seated at a comfortable distance from the computer screen. The background questionnaire was completed at the end of the testing session.

#### Statistical Procedures

Participant performance was compared by using different independent sample *T*-tests, ANOVAs and Bayesian ANOVAs. An additional chi-square test was also employed in order to compare the three language groups (Love et al., 2015).

Bayesian ANOVAs were used given current critiques regarding inferential statistics related to *p*-values, confidence intervals, and null hypotheses (Wagenmakers, 2007; Wagenmakers et al., 2015). This type of analysis is based on the comparison of two competing models, i.e., the null and the alternative model. The null model stipulates that only a null value may be possible (no group effect exists), while the alternative model argues that an alternative model may be accepted (group effects exist).

Bayesian inference is based on the computation of the most probable model given data from the Bayes factor. Despite no clear consensus, a Bayes factor of one has been suggested to reflect no evidence, between 1 and 3 anecdotal evidence, and between 3 and 10 substantial evidence (Lee and Wagenmakers, 2014).

## Results

### Language and General Control Measures

Different general control variables were assessed to ensure the comparability of the three tested language groups (HFLSs, LFLSs, and monolinguals). These variables included age, socio-cultural status, non-verbal intelligence skills, video game practice, L1 and L2 receptive vocabulary skills, L1 and L2 productive vocabulary skills, L1 and L2 general vocabulary skills, L1 and L2 self-estimated proficiency levels, as well as L1 and L2 self-estimated frequency use. HFLSs and LFLSs were additionally assessed in terms of self-estimated L3 proficiency levels and L3 self-estimated frequency use.

Chi-square tests revealed no significant group difference in terms of gender, χ^2^ (2) = 1.04, *p* = 0.59. The ANOVAs analysis showed no group effects on age [*F*(2,76) = 1.38, *p* = 0.25], SES level [*F*(2,76) = 0.09, *p* = 0.90], non-verbal intelligence skills [*F*(2,76) = 1.71, *p* = 0.18], video game practice [*F*(2,76) = 1.46, *p* = 0.23], L1 receptive vocabulary skills [*F*(2,76) = 1.54, *p* = 0.22], L1 productive vocabulary skills [*F*(2,76) = 0.57, *p* = 0.56], L1 general vocabulary skills (LexTALE) [*F*(2,76) = 0.16, *p* = 0.84], and L1 self-estimated levels of proficiency [*F*(2,76) = 1.32, *p* = 0.27]. T-tests showed no significant group difference between HFLSs and LFLSs in terms of L2 receptive vocabulary skills, *t*(49) = −0.34, *p* = 0.73; L2 productive vocabulary skills, *t*(49) = 0.66, *p* = 0.51); L2 general vocabulary skills (LexTALE), *t*(49) = 1.43, *p* = 0.15; L2 self-estimated level of proficiency, *t*(49) = 1.52, *p* = 0.13; and L2 frequency of use, *t*(49) = 0.21, *p* = 0.83. Results also showed no significant group difference between HFLSs and LFLSs in terms of L3 self-estimated level of proficiency, *t*(49) = −1.20, *p* = 0.23, and L3 frequency of use, *t*(49) = −0.04, *p* = 0.96. Descriptive statistics are presented in Table 1.

**TABLE 1:** Descriptive statistics in age, SES, non-verbal intelligence, video-game practice, first language (L1) receptive vocabulary skills, L1 productive vocabulary skills, L1 general vocabulary skills, L1 self-estimated proficiency level, L1 self-estimated frequency use, second language (L2) receptive vocabulary skills, L2 productive vocabulary skills, L2 general vocabulary skills, L2 self-estimated proficiency level, L2 self-estimated frequency use, L3 self-estimated proficiency level, and L3 self-estimated frequency use.

	**High-frequency switchers *N* = 30**	**Low-frequency switchers *N* = 21**	**Monolinguals *N* = 28**
	Mean (SD)	Mean (SD)	Mean (SD)
Age (years)	25.73 (6.08)	24.90 (6.65)	27.89 (7.16)
Socio-cultural status (years of education)	15.63 (2.78)	15.38 (2.97)	15.39 (1.03)
Non-verbal intelligence (60/60)	52.13 (3.35)	52.05 (3.82)	50.11 (6.00)
Video game practice (hours per week)	0.70 (1.93)	0.21 (0.60)	0.17 (0.54)
L1 receptive vocabulary skills (z scores)	0.89 (0.36)	0.97 (0.32)	1.04 (0.26)
L1 productive vocabulary skills (total correct responses)	144.3 (26.46)	149.8 (23.16)	142 (26.32)
L1 general vocabulary skills (%) (LexTALE)	88.46 (6.06)	88.80 (8.08)	87.05 (17.12)
L1 self-estimated proficiency level (24/24)	23.00 (2.03)	23 (1.26)	22.29 (2.03)
L1 daily self-estimated frequency use (%)	57.08 (22.39)	58.74 (28.96)	99.63 (1.46)
L2 receptive vocabulary skills (z scores)	0.48 (0.52)	0.53 (0.54)	−4.23 (1.07)
L2 productive vocabulary skills (total correct responses)	117.7 (33.49)	112.1 (22.75)	
L2 general vocabulary skills (%)	81.79 (8.95)	78.33 (7.67)	64.42 (6.30)
L2 self-estimated proficiency level (24/24)	21.00 (2.77)	19.81 (2.69)	9.28 (2.66)
L2 daily self-estimated frequency use (%)	36.64 (19.68)	35.25 (27.13)	0.36 (1.49)
L3 self-estimated proficiency level (24/24)	15.10 (4.58)	16.45 (2.82)	
L3 daily self-estimated frequency use (%)	5.77 (10.57)	5.89 (7.90)	

### Experimental Measures

Both response times and accuracy measures were analyzed. For the cognitive flexibility task, an ANOVA carried on response time revealed a significant group effect [*F*(2,76) = 5.93, *p* < 0.005; $\eta_{\text{p}}^{2}$ = 0.13]. Further *post hoc* analysis (Tukey correction) showed that HFLSs exhibited a faster response time as compared to LFLSs and monolinguals [HFLSs vs LFLSs: *t*(49) = −3.23, *p* < 0.05, *d* = −1.00; HFLSs vs monolinguals: *t*(56) = −2.50, *p* < 0.05, *d* = −0.66]. However, no significant group difference was observed between LFLSs and monolinguals in this respect: t(48) = 0.91, *p* = 0.63. For the cognitive flexibility task, the Bayesian factor on response time revealed that alternative models that included a group effect were over 10 times more likely than the null model to include no group effect (BF10 = 10.10). A *post hoc* test revealed that the model that included a significant group difference between HFLSs and LFLSs was over 33 times more likely as compared to the null model including no group difference (BF10 = 33.49). As to differences between HFLSs and monolinguals, the alternative model was over three times more likely as compared to the null model comprising no group difference (BF10 = 3.46). The alternative model, however, did not support a significant group difference between LFLSs and monolinguals (BF10 = 0.38). Moreover, the null model (sustaining no group difference) for this task was 0.09. No significant speed–accuracy trade-off was observed between response speed and error rates as shown by a correlation analysis conducted between the two (*r* = 0.20; *p* = 0.07). This result was also confirmed by Bayesian correlations, which showed that the alternative model (supporting a significant correlation between response time and error rates) was only 0.68 (*r* = 0.20; BF10 = 0.66).

An additional correlation analysis was conducted for the cognitive flexibility task between task response times and language-switching rates for participants tested during the present study (HFLSs and LFLSs). Results revealed a significant correlation between the two measures: *r* = −0.31; *p* < 0.01. These results were confirmed by a Bayesian correlation analysis which showed similar patterns of results: *r* = −0.31; BF10 = 6.69. These findings suggest that the alternative model supporting a significant correlation is six times more likely as compared to the null model supporting no correlation. Language-switching frequency and cognitive flexibility skills and to potentially highlight evidence which could straighten the argument that frequent language switching enhances cognitive flexibility skills, we further conducted an additional correlation between language-switching rates and response times for all the initial cohort of proficient bilinguals tested (*N* = 68) prior to establishing the two groups of high and low language switchers. Inferential correlation analysis revealed a significant link between these measures: *r* = −0.31; *p* < 0.05. These results were also confirmed by a Bayesian correlation analysis: *r* = −0.31; BF_10_ = 7.81. This hypothesis was based on a prior negative hypothesis (negative correlation between response times and language-switching frequency). These findings indicate that language-switching frequency is directly linked to cognitive flexibility skills.

Concerning the alertness task, the ANOVA analysis revealed no significant group effects on response time [*F*(2,76) = 0.49, *p* = 0.61; $\eta_{\text{p}}^{2}$ = 0.01]. For this task, the Bayes factor was only 0.16 for response time. Moreover, the null model (supporting no group difference) for this task was 6.20. *Given no significant group effects observed on this task, no further correlation analysis was conducted on the initial cohort of proficient bilinguals tested.*

A similar pattern was also observed for the response inhibition task, with no significant group effect on response time [*F*(2,76) = 0.79, *p* = 0.45; $\eta_{\text{p}}^{2}$ = 0.02]. For this task, the Bayes factor for the alternative model (supporting a group difference) was only 0.20 for response time. Moreover, the null model (sustaining no group difference) for this task was 4.94. *No further correlation analysis was further conducted, given no significant group effects observed on this task on the initial cohort of proficient bilinguals tested.*

A series of ANOVAs was conducted on accuracy responses for cognitive flexibility, alertness, and response inhibition tasks.

No significant differences were determined on the cognitive flexibility task [*F*(2,76) = 0.51, *p* = 0.59; $\eta_{\text{p}}^{2}$ = 0.01; mean for low-switching bilinguals: 2.52, SD: 3.76; range for low-switching bilinguals: 0–17 errors per 100 items; mean for high-switching bilinguals: 2.13, SD: 1.96; range for high-switching bilinguals: 0–6 errors per 100 items; mean for monolinguals: 1.78, SD: 1.81; range for monolinguals: 0–7 errors per 100 items]. Concerning errors made on this task, the Bayesian analysis showed that the alternative model was only 0.16.

The alertness task showed no significant group differences [*F*(2,76) = 1.58, *p* = 0.21; $\eta_{\text{p}}^{2}$ = 0.04; mean for low-switching bilinguals: 0.42, SD: 0.50; range for low-switching bilinguals: 0–1 errors per 18 items; mean for high-switching bilinguals: 0.66, SD: 0.47; range for high-switching bilinguals: 0–1 errors per 18 items; mean for monolinguals: 0.50, SD: 0.50; range for monolinguals: 0–1 errors per 18 items]. For this task, the Bayes factor was only 0.37 for errors.

The response inhibition task showed no significant differences: [*F*(2,76) = 2.33, *p* = 0.10; $\eta_{\text{p}}^{2}$ = 0.05; mean for low-switching bilinguals: 0.52, SD: 0.87; range for low-switching bilinguals: 0–3 errors per 20 items; mean for high-switching bilinguals: 0.96, SD: 0.99; range for high-switching bilinguals: 0–4 errors per 20 items; mean for monolinguals: 0.50, SD: 0.83; range for monolinguals: 0–3 errors per 20 items]. For this task, the Bayes factor was only 0.68 for error rates.

These results seem to confirm that oral language-switching frequency does have a positive effect on cognitive flexibility skills in proficient bilingual adults. These findings, however, offer no significant evidence for a positive effect of oral language-switching frequency on alertness and response inhibition. Descriptive statistics, mean comparisons using inferential and Bayesian statistics for measures of alertness, response inhibition, and cognitive flexibility (reaction times in milliseconds and errors) are presented in Table 2.

**TABLE 2:** Descriptive statistics, mean comparisons by using inferential and Bayesian statistics in measures of alertness, response inhibition, and cognitive flexibility (reaction times in milliseconds and errors).

				**Inferential statistics**		**Bayesian statistics**			
				
	**High-frequency switchers *N* = 30**	**Low-frequency switchers *N* = 21**	**Monolinguals *N* = 28**	**Group effect *p***	**Chi-squared test**	**BF_10_**	**BF_10_ (error %)**	**BF_01_**	**BF_01_ (error %)**
							
	**Mean (SD)**	**Mean (SD)**	**Mean (SD)**						
Alertness RT (ms)	238.6 (28.56)	248.3 (48.31)	238.4 (40.44)	0.61	0.01	0.161	0.028	6.204	0.028
Alertness Errors (max = 18)	0.66 (0.47)	0.42 (0.50)	0.50 (0.50)	0.21	0.04	0.379	0.035	2.636	0.035
Response inhibition RT (ms)	383.9 (65.44)	405.7 (70.42)	387.8 (54.63)	0.45	0.02	0.202	0.030	4.945	0.030
Response inhibition Errors (max = 20)	0.96 (0.99)	0.52 (0.87)	0.50 (0.83)	0.10	0.05	0.688	0.022	1.453	0.022
Cognitive flexibility RT (ms)	531.3 (104.3)	645.1 (125.2)	612.6 (140.6)	0.00	0.13	10.106	0.016	0.099	0.016
Cognitive flexibility Errors (max = 100)	2.13 (1.96)	2.52 (3.76)	1.78 (1.81)	0.59	0.01	0.165	0.028	6.074	0.028

## Discussion

Barbu et al. (2018) have recently attempted to assess the effect of language-switching frequency on attentional and executive functioning (alertness, response inhibition, and cognitive flexibility) in proficient bilinguals. Their results revealed a small positive group difference (*p* = 0.03), with HFLSs exhibiting faster responses as compared to LFLSs on a cognitive flexibility task. However, no significant group differences were observed on tasks assessing alertness or response inhibition. The authors suggested that these results might be explained by the fact that the tasks used to assess these skills did not require a behavior similar to language switching, i.e., switching between mental sets. The group difference observed on the cognitive task was quite small, which might be attributed to the different language backgrounds (different types of L1–L2 pairs) of HFLSs and LFLSs bilinguals tested.

The aim of the present study was to replicate Barbu et al.’s (2018) study by assessing bilingual HFLSs and LFLSs adults with homogenous language backgrounds, i.e., German- and French-speaking bilinguals, and to compare the performance of these two groups to the monolingual control group in order to determine if bilingualism in itself has a positive impact on alertness, response inhibition, and cognitive flexibility.

The results of the present study revealed that HFLSs showed faster responses as compared to LFLSs and monolinguals on the cognitive flexibility task. No significant group difference was observed, however, in this respect between LFLSs and monolinguals. No significant group differences were seen between HFLSs and LFLSs and monolinguals on tasks assessing alertness and response inhibition. Not observing significant group differences on accuracy measures might be an indicator that participants exhibited good task performance, confirming that they were competent and suggesting that the advantage of language-switching frequency would be reflected only in time measures. The present results replicate Barbu et al.’s (2018) and Prior and Gollan’s (2011) findings showing that language-switching frequency has a positive effect on general switching or cognitive flexibility skills. This outcome might be explained by the fact that the cognitive flexibility task used to assess these skills requires switching skills or the ability to shift between different items or mental sets and to classify items according to their specific abstract category (letter and number in the present case). This process is similar to language switching, in which constant toggling between language sets and item categorizations is required. Language-switching frequency, rather than bilingualism *per se*, seems to explain the significant group advantage observed in HFLSs as compared to LFLSs on the cognitive flexibility task given that these groups were comparable on L1, L2, and L3 proficiency levels and frequencies of language use. Furthermore, if bilingualism had an impact on cognitive flexibility skills, not only HFLSs but also LFLSs would have outperformed monolinguals on the cognitive flexibility task, which was not the case. Globally, these results suggest that language-switching frequency and not bilingualism *per se* might be a specific underlying factor in cognitive flexibility skills in proficient bilinguals.

The lack of differences between HFLSs and LFLSs in tasks assessing alertness and response inhibition confirms our previous results showing that language-switching frequency does not impact these functions (Barbu et al., 2018). Furthermore, our findings suggest that bilingualism *per se* does not enhance alertness and response inhibition skills, as no significant group differences were revealed between LFLSs and monolinguals on tasks assessing these skills. These results, however, do not align with Costa et al.’s (2008) findings showing that bilingualism enhances alertness skills. In this study, the authors used the Attention Network Test (ANT; Fan et al., 2002) in order to assess alertness, monitoring, and interference inhibition skills. During this task, participants are presented with different arrows presented on-screen and asked to indicate the position of the central arrow (all arrows pointing in the same direction: congruent condition; the central arrow (target) pointing in the opposite direction as compared to the flankers: incongruent condition). The difference in response speed between congruent and incongruent conditions has been indexed as a conflict resolution effect. Alertness has been studied by the presentation of a cue before the target stimulus, presumably argued to enhance responses (trials accompanied by a cue as compared to trials where no cue is present). Finally, the orienting network was studied by presenting a cue that signals the position on-screen where the target item will appear. Results revealed that proficient Catalan–Spanish bilinguals exhibited better alertness, monitoring, and conflict resolution performance as compared to monolingual peers. Positive effects of bilingualism on alertness skills have also been observed by using a similar version of the ANT task (Costa et al., 2009), which included, however, a higher level of monitoring conditions (higher number of incongruent trials requiring conflict resolution skills). This advantage was attributed to the improved ability of proficient bilinguals to resolve the inherent conflict during language selection. Authors argued that these advantages are likely to be due to language-switching frequency, despite that this behavior was not controlled for. Given that participants were proficient bilinguals who lived in a bilingual community (Catalonia), they were probably switching often between languages. In the present study, we used a different alertness task with more simple requirements (no facilitating salient cue) than the ANT used by Costa and colleagues. Differences in task design and complexity might be the reason for which we found no significant effect on the alerting task. Future studies should involve the use of multiple conditions and salient cues when assessing the effects of language-switching frequency and bilingualism on alertness skills.

Concerning results obtained on the response inhibition task, some studies have shown positive effects of bilingualism on response inhibition (e.g., Fernandez et al., 2013), while others have not (e.g., Moreno et al., 2014). All these authors have, however, used different tasks in order to assess response inhibition, which might explain the inconsistent findings. Fernandez et al. (2013) assessed inhibitory skills in Spanish–English bilinguals and English monolinguals by using a non-linguistic auditory Go/NoGo task which measured behavioral and neural responses (event-related potential—N200). During this task, participants were required to press a response button on target tone pairs (Go trials) and withhold their responses on non-target trials (NoGo trials). NoGo trials which required inhibition of non-desired automatic responses were indexed as an inhibition marker. Results revealed no significant group differences at a behavioral level on either errors rates or response speed. At a neural level, however, results revealed greater mean amplitude for N200 in bilinguals as compared to monolinguals, suggesting that bilinguals were more able to mobilize their inhibitory resources as compared to monolinguals when inhibiting automatic NoGo responses. The authors conducted a subsequent study (Fernandez et al., 2014) in which they extended these findings to a visual Go/NoGo task. Results replicated results for the auditory task (greater mean N200 amplitude for NoGo trials). For the visual Go/NoGo task, however, event-related brain potentials did not distinguish between bilinguals and monolinguals either behaviorally or neurally. These results do not align, however, with Moreno et al.’s (2014) results, which observed neural advantages (higher neural activation for the N200 wave form) on a visual Go/NoGo task in bilinguals as compared to monolinguals. Note, however, that Fernandez et al. (2014) and Moreno et al. (2014) used different task designs when assessing response inhibition, which might explain the inconsistent results. Our results converge with those of Moreno et al. (2014) and show no positive behavioral effects of bilingualism or language-switching frequency on a visual Go/NoGo task assessing response inhibition. Our results are in line with previous findings showing that bilingualism does not impact response inhibition as opposed to interference suppression (e.g., Luk et al., 2010). Given this, it might be that positive effects of bilingualism or language-switching frequency on *response inhibition* are more likely to be observed in auditory rather that in visual inhibition tasks.

These findings also suggest that positive effects of bilingualism might be easier to observe at a neural level. In this sense, *brain imaging measures such as EEG, (f)MRI, and/or MEG* might offer more detailed information concerning the effects of bilingualism but also language-switching frequency on attentional and executive functioning and might be *more* appropriate measures to confirm the observed findings (absence of a positive effect of language-switching frequency and bilingualism on alertness and response inhibition).

A strength of the present study is the control of several in-between variables likely to influence performance on executive tasks such as language-switching frequency or L2 mastery and use. Individual differences in language-switching experience, frequency of L2 use, or degree of L2 mastery have indeed been suggested to modulate outcomes and to explain the inconsistency between current findings regarding the impact of bilingualism on executives functioning (for a systematic review, see de Bruin, 2019). Bilingualism related experiences are indeed not the same, and these variations are mostly likely to impact results. For instance two bilinguals, despite speaking the same two languages and mastering the two to the same degree, can still differ tremendously in how they use their two languages in their daily lives. In order to understand what about bilingualism is really responsible for advantages on executive functioning, a detailed description of bilingual language experiences should be provided by future studies. These individual differences should be automatically measured when assessing bilinguals. This also implies that we should consider bilingualism as a continuum with all these variables taken together instead of having to set arbitrary boundaries on bilingual experiences. We can, however, agree that providing a detailed, complete, and objective assessment of bilingual language experience and profiles can be rather challenging.

In conclusion, the results of the present study seem to confirm that language-switching frequency represents an underlying factor of the improved cognitive flexibility skills in proficient bilingual adults. These findings highlight the importance of taking into account this linguistic factor in bilingual research. Our findings also suggest that neither language-switching frequency nor bilingualism *per se* improves alertness or response inhibition skills. For future considerations, tasks previously shown to exhibit positive effects of bilingualism (e.g., Costa et al., 2008; Costa et al., 2009) should be applied on HFLSs, LFLSs, and monolinguals in order to establish if the positive effects put forward are due to bilingualism *per se* or to language-switching frequency.

## Data Availability Statement

The data sets generated for this study are available on request to the corresponding author.

## Ethics Statement

This research was approved by the ethics committee of the Faculty of Psychology and Educational Sciences (University of Liège, Belgium). At that moment (2012) the president of the ethics commitee was Isabelle Hansez (Director of the Valorisation of Human Resources Unit, Faculty of Psychology and Educational Sciences, University of Liège). The patients/participants provided their written informed consent to participate in this study.

## Author Contributions

MP: conceptualization of the study, data treatment, and conceiving and writing the article. SG: establishing methodology, collecting and analyzing the data, giving final feed-back before submission. All authors contributed to the article and approved the submitted version.

## Conflict of Interest

The authors declare that the research was conducted in the absence of any commercial or financial relationships that could be construed as a potential conflict of interest.
